# Abnormal circadian rhythm and cortisol excretion in autistic children: a clinical study

**DOI:** 10.3325/cmj.2013.54.33

**Published:** 2013-02

**Authors:** Malarveni Damodaran Lakshmi Priya, Arumugam Geetha, Vijayashankar Suganya, Sridharan Sujatha

**Affiliations:** 1 Department of Biochemistry, Bharathi Women’s College, University of Madras, Chennai, Tamil Nadu, India; 2 Madras Medical College and Pediatrician Institute of Child Health and Hospital for Children, Chennai, Tamil Nadu, India

## Abstract

**Aim:**

To determine the circadian rhythm alteration of cortisol excretion and the level of corticosteroids in children with different grades of autism severity.

**Methods:**

The study included 45 children with different grades of autism severity (low [LFA], medium [MFA], and high functioning autism [HFA]), 15 in each group, and 45 age/sex-matched children with typical development. The urinary levels of free cortisol (at three phases of 24-hour cycle), corticosteroids, vanilylmandelic acid, and 5-hydroxyindole acetic acid were determined.

**Results:**

Alteration in the pattern of cortisol excretion (Phases I, II, and III) was observed in children with LFA (Phase I: 43.8 ± 4.43 vs 74.30±8.62, *P* = 0.000; Phase II: 21.1±2.87 vs 62±7.68, *P* < 0.001; Phase III: 9.9 ± 1.20 vs 40 ± 5.73, *P* < 0.001) and MFA (Phase I: 43.8 ± 4.43 vs 52.6±7.90, *P* < 0.001; Phase II: 21.1±2.87 vs 27.4±4.05, *P* < 0.001; Phase III: 9.9 ± 1.20 vs 19 ± 2.50, *P* < 0.001) compared to the control group. The corticosteroids excretion levels were higher in all the groups of children with autism than in the control group. The level of 5-hydroxyindole acetic acid was significantly higher in children with LFA (8.2±1.48 vs 6.8±0.85, *P* < 0.001) and MFA (8.2±1.48 vs 7.4± 0.89, *P* = 0.001) and not significantly higher in children with HFA than in the control group. The changes were correlated with degrees of severity of the disorder.

**Conclusion:**

These data suggest that altered cortisol excretion pattern and high level of corticosteroids in urine may probably be a consequence of altered hypothalamic-pituitary-adrenal axis function, which may contribute to the pathogenesis and affect the severity of autism.

Autistic disorder is a behaviorally defined neurodevelopmental disorder characterized by deficits in social interaction, language, and communication, and by repetitive behaviors, which manifest in early postnatal life (1). It belongs to the spectrum of closely related conditions also referred to as autism spectrum disorders (ASDs). The incidence of ASDs has increased significantly over the last decades and is currently 1 in 150, affecting boys four times more often than girls (2). Autism remains one of the very few conditions classified as a syndrome, defined only in terms of observable symptoms (3), largely because no accepted biochemical diagnostic markers are currently available. Research into the pathophysiology and etiology of autistic disorder has been ongoing for nearly half a century and still the cause remains unknown.

Circadian rhythm is controlled by a “biological clock,” a time keeping system (24 hours) in the organism. Biological clock also provides internal temporal organization and ensures that internal changes take place in coordination with one another. Although circadian rhythm is synchronized with light and dark cycle, it is also affected by psychological (defective adaptive capacity to environmental demand) and physical factors (hypoglycemia, infection), as well as stress. In people lacking a proper circadian rhythm, biological clock ceases to function normally resulting in defective physiological ability to respond to the demands of the environment (4). Experimental studies have investigated different types of stress and their effects on the hypothalamic-pituitary-adrenal (HPA) axis (5). HPA axis is altered significantly in mood disorders and functional illness, including anxiety disorders, bipolar disorder, insomnia, posttraumatic stress disorder, border line personality disorder, and attention deficit hyperactivity disorder (ADHD) (6). Dysregulation of biological systems, including the HPA axis, has been also observed in autism (7). Autism has often been characterized as a disorder accompanied by increased arousal, stress, and sensory sensitivity. In research on autism, HPA axis deserves special attention, since it is the basis for emotions and social interactions (8,9).

The HPA axis has a well-characterized circadian pattern. Under the influence of stress, this pattern is altered and homeostasis of stress-related neuroendocrine function is disrupted, with adverse impact on health. Essential to this stress response is the activation of the HPA system, resulting in the release of glucocorticoid hormones from the adrenal cortex. The primary glucocorticoid in humans is cortisol (hydrocortisone), which exhibits diurnal variations peaking in the early morning hours (about 30 minutes after waking), declining rapidly in the morning, decreasing slowly in the afternoon, and reaching its lowest level in the evening. This circadian rhythmic release of cortisol can be well studied by the level of its excretion in urine in different time intervals during the day. This is a well developed pattern already in the third month of infancy (10).

Impaired immune functions and disturbed circadian rhythm in ASDs may be due to aberrations in fatty acid metabolism, particularly the eicosanoid production (11,12), which plays a major role in neuronal development and behavior, learning ability, and memory (13).

Prostaglandin E_2_ (dinoprostone) is an important arachidonic acid metabolite formed by the action of cyclooxygenase-1. Prostaglandin E_2_ is an important signaling molecule involved in pain or synaptic plasticity in the nervous system. Along with free radicals, prostaglandin metabolites have been shown to influence the pathological disturbance in nervous system (14). The level of cortisol and its catabolic excretable compounds in urine are a measure of their metabolic turnover and used to evaluate the neurodevelopmental complications.

Therefore, the present study aims to evaluate the alteration in the circadian rhythmic pattern of cortisol by measuring its excretion in different time intervals during the day and also to determine the level of excretion of adrenal cortex hormones (11- hydroxy corticosteroids, 17- oxogenic steroids, and total 17- hydroxycorticosteroids), along with vanilyl mandelic acid, 5-hydroxyindoleacetic acid, and prostaglandin E2, which could also shed more light on the pathogenesis of autism and its severity.

## Study design and methods

### Patients

The study included 45 children with autism attending the school for children with special needs Aikya, Maruti Seva at Chennai, Tamil Nadu, India from 2010-2012. The institution used Check of Autism in Toddlers (15) to assess autism. Autistic children were classified according to the method adopted from Childhood Autism Rating Scale (CARS) (16) as those with low functioning autism (LFA), medium functioning autism (MFA), and high functioning autism (HFA). Each group comprised 15 children. Forty-five age- and sex-matched healthy children were used as controls. They were attending regular school at the same location and had no history of neurodevelopmental complications. The boys and girls ratio was 4:1, and they were 4-12 years old (Table 1).

**Table 1:** Clinical history of children with autism and children with typical development*

Characteristics	Autism		Typical development
**Number of children**	45 (15 in each group)		45
**CARS value (15-60)**	LFA = 46-60; MFA = 31-45; HFA = 15-30		<10
**Male/female ratio**	36/9		36/9
**Age in years, range**	4-12		4-12
**Children with gluten sensitivity**	LFA = 12/15; MFA = 10/15; HFA = 5/15		0
**Economic status of the parents**	High (above Rs. 4 lakhs)	LFA = 2; MFA = 1; HFA = 2	High = 5; Medium = 38; Low = 2
	Medium (- Rs. 2.5 lakhs to Rs. 4 lakhs)	LFA = 13; MFA = 12; HFA = 13	
	Low (Rs. 60 000 to 2.5 lakhs)	LFA = 0; MFA = 2; HFA = NIL	
**Nutritional status of the children^†^**	Good	LFA = 0; MFA = 0; HFA = 2	Good = 45
	Better	LFA = 2; MFA = 4; HFA = 2	
	Poor	LFA = 13; MFA = 11; HFA = 11	
**Children with special talents (dancing, humming, drawing, assembling puzzles)^‡^**	LFA = 3/15; MFA = 7/15; HFA = 11/15		/
**No. of children with low muscle tone^§^**	LFA = 14/15; MFA = 12/15; HFA = 11/15		0
**No. of children with ear infection during the study period**	LFA = 2/15; MFA = 0; HFA = 0		0
**No. of children with sleep disturbance^║^**	LFA = 13/15; MFA = 11/15; HFA = 6/15		0
**No. of children with mood disorder^¶^**	LFA = 11/15; MFA = 9/15; HFA = 6/15		0
**No. of children on antibiotic treatment**	0		0
No. of children with gastrointestinal problems**	LFA = 9/15; MFA = 6/15; HFA = 0		0
**No. of parents given their cooperation**^††^	40/45		45
**No. of parents appreciated the study**^‡‡^	38/45		45

*Abbreviations: LFA – low functioning autism; MFA – moderately functioning autism; HFA – high functioning autism, CARS – Childhood Autism Rating Scale.

†Assessed in terms physical examination including weight, stature, head circumference, and arm measurements. These data were obtained from the children’s clinical histories kept at the school.

‡The children had talents in general but a few autistic children had a very unique and exemplary way of displaying their talents.

**§**Muscle tone was determined by squeezing the muscle to feel resistance to compression and lifting up and moving the limbs and feeling the resistance to the movement. These data were obtained from the children’s clinical histories kept at the school.

**║**Sleep disturbance mainly refers the discontinuous sleep pattern, due to frequent bed-wetting or sudden waking up in fear, as observed by the parents.

**¶**Mood swings in school, including non cooperation with the care takers and sudden screaming.

**Frequent diarrhea, stomach ache, or gut dysbiosis.

††The parents volunteered in giving the samples of their children. They also helped us in follow up studies.

‡‡The parents found the subject interesting and were satisfied with the outcome of the study.

### CARS classification

CARS classification is a 15-item scale that identifies children with autism and distinguishes them from other children with compromised development but without autism (16). It also differentiates mild to moderate from severe autism (17). It is brief and appropriate for children older than 2 years. The scale evaluates behavior in 14 domains that are generally affected in autism, plus a single category for general impression of autism (18). These 15 items are as follows: relating to people, imitation, emotional response, body use, object use, adaptation to change, visual response, listening response, taste, smell, and touch response and use, fear or nervousness, verbal communication, nonverbal communication, activity level, level and consistency of intellectual response, and general impressions. The scores assigned to each domain vary from 1 (within the limits of normality) to 4 (severe autistic symptoms). The total score varies from 15 to 60 and the cutoff point for autism is 30 (16).

### Collection of urine sample

Parents collected urine samples from children at 3 different time points as follows: 9.00 pm to 7.00 am (morning sample – Phase I), 7.00 am to 2.00 pm (noon sample – Phase II), and 2.00 pm to 9.00 pm (evening sample – Phase III) in 3 different sterilized containers labeled accordingly. After taking 25 µL-50 µL for determination of cortisol, the samples were poured and collected in a 24-hour container for further assays. On receipt of the specimen at the laboratory, the volume was noted, and after vigorous shaking, stored at -4°C with 3-4 drops/100 mL of formalin as preservative until subsequent analysis. Urine samples were collected on three consecutive days and analyzed separately, and the average values for each sample was calculated. The study protocol was approved by the Institutional Ethics Committee, Madras Medical College, Chennai – 3, EC No. 22072012.

### Methods

The fluorimetric method developed by Mattingly et al (19) was used for the determination of urinary 11-hydroxycorticosteroids against cortisol as the standard. Neutral 17- oxosteroids level was determined by the method of Norymberski et al (20), which used ethylene dichloride to extract the steroids, and tetramethyl ammonium hydroxide (25% w/v aqueous solution) was used as alkali in the color development and was read colorimetrically at 520 nm using a green filter. For the determination of total 17-oxosteroids (20), sodium bismuthate was used for oxidation and 12% sodium metabisulphite was used to reduce the bismuthate. The color was developed by Zimmerman reaction similar to 17-oxosteroids and the color developed was read colorimetrically at 520 nm using a green filter. 17-oxogenic steroids level was determined using the formula:

17-oxogenic steroids = total 17-oxosteroids - neutral 17-oxosteroids

Determination of total 17-hydroxycorticosteroids was done using metaperiodate by the method of Few (21). The level of urinary free cortisol was determined by EIAgen cortisol kit (ADALTIS, Rome, Italy) using Elisa strip reader catalog no: LI4003K.

Vanilyl mandelic acid was measured using UV-spectrometer by the method of Pisano et al (22). We used a more rigorous method by Udenfriend et al, which however, only determines 5-HIAA (23). The urine was first treated with dinitrophenyl hydrazine to react with ketoacids, which may interfere later. Any indole acetic acid present was extracted into chloroform. After saturation with sodium chloride, the hydroxyindole acetic acid was extracted into ether and then returned to phosphate buffer pH 7.0 for colorimetric assay. Prostaglandin E_2_ was determined using a kit purchased from Arbor Assays (*www.arborassays.com*), catalog no: K018-H1.

### Statistical analysis

The results are presented as mean ± standard deviations. The groups were compared with one-way ANOVA with *post-hoc* Bonferroni test, and the *P* value <0.05 was considered as significant. The parameters were also analyzed by Spearman rank test, and r_s_ values were calculated to find the significance of correlation.

## Results

**Figure 1:**
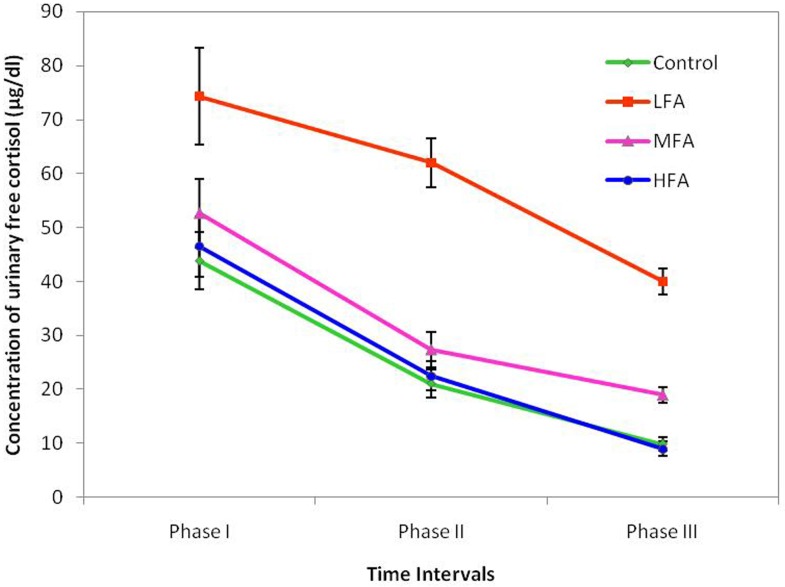
Level of urinary free cortisol (mean±standard deviation) collected in three different time intervals in children with low functioning autism (LFA), medium functioning autism (MFA), and high functioning autism (HFA) and age and sex-matched children with typical development. *P*<0.001 (control vs LFA); *P*<0.01 (control vs MFA); *P<*0.05 (control vs MFA and HFA); non-significant (control vs HFA); *P*<0.001 (LFA vs HFA).

The level of excretion of free cortisol in the LFA (Phase I: 43.8 ± 4.43 vs 74.30**±**8.62, 95% confidence interval [CI] 69.94-78.66; *P* < 0.001; Phase II: 21.1**±**2.87 vs 62**±**7.68, 95% CI 58.11-65.89, *P* < 0.001; Phase III: 9.9 ± 1.20 vs 40 ± 5.73, 95% CI 37.1-42.9, *P* < 0.001, *post-hoc* Bonferroni test) and MFA group (Phase I: 43.8 ± 4.43 vs 52.6**±**7.90, 95% CI 48.6-56.6, *P* < 0.001; Phase II: 21.1**±**2.87 vs 27.4**±**4.05, 95% CI 25.35-29.45, *P* < 0.001; Phase III: 9.9 ± 1.20 vs 19 ± 2.50, 95% CI 17.73-20.27, *P* < 0.001, *post-hoc* Bonferroni test) was significantly higher than in the control group (Figure 1). Excretion of cortisol in HFA group showed no significant alteration from the control group (Phase I: 43.8 ± 4.43 vs 46.5 ± 6.60, 95% CI 43.16-49.84 *P* = 0.179; Phase II: 21.1 ± 2.87 vs 22.5 ± 2.52, 95% CI 21.22-23.78 *P* = 0.352; Phase III: 9.9 ± 1.20 vs 9.0 ± 0.97, 95% CI 8.51-9.49 *P* = 0.177, *post-hoc* Bonferroni test) but showed a highly significant alteration at the phases I (74.3 ± 8.62 vs 46.5 ± 6.60, *P* < 0.001), II (62 ± 7.68 vs 22.5 ± 2.52, *P* < 0.001), and III (40 ± 5.73 vs 9 ± 0.97, *P* < 0.001) when compared to LFA group. The cortisol excretion in the autistic groups had a significant positive correlation with the severity of autism (Table 2).

**Table 2:** Rank correlation between CARS and corticosteroids, free cortisol, vanilyl mandelic acid, and prostaglandin E_2_ in different groups of autistic children (n = 45)*

Elements	rs^†^	*P*
**11- Hydroxycorticosteroids vs CARS**	+0.795	<0.001
**Neutral 17- oxosteroids vs CARS**	+0.852	<0.001
**Total 17- oxosteroids vs CARS**	+0.902	<0.001
**17- Oxogenic steroids vs CARS**	+0.856	<0.001
**Total 17- hydroxycorticosteroids vs CARS**	+0.799	<0.001
**Free cortisol vs CARS** **(Phase I)**	+0.899	<0.001
**Free cortisol vs CARS** **(Phase II)**	+0.801	<0.001
**Free cortisol vs CARS** **(Phase III)**	+0.787	<0.001
**Vanilyl mandelic acid vs CARS**	+0.834	<0.001
**Prostaglandin E_2_ vs CARS**	+0.876	<0.001

*CARS – Childhood Autism Rating Scale.

†Based on the critical values of the rank correlation (Spearman rho’s), null hypothesis of no correlation was rejected and it was concluded that the level of corticosteroids, cortisol, vanilyl mandelic acid, and prostaglandin E_2_ correlated with the severity of autism.

11-hydroxycorticosteroids, neutral 17-oxosteroids, total 17-oxosteroids, 17-oxogenic steroids, and total 17-hydroxycorticosteroids showed a significantly greater excretion in all groups of autistic children (Table 3). The level of excretion of vanilyl mandelic acid was also significantly higher (*P* < 0.001) in autistic children than in the control group. The level of excretion of 5-hydroxyindole acetic acid was significantly higher in LFA (6.8 ± 0.85, *P* < 0.001) and in MFA (7.4 ± 0.89, *P* = 0.001) and non-significantly higher in HFA (7.80 ± 0.98, *P* = 0.277) when compared to the control group (8.2 ± 1.48). The level of prostaglandin E_2_ in autistic children was higher (LFA = 11.41 ± 1.65; *P* < 0.001, MFA = 8.02 ± 1.12; *P* < 0.001, HFA = 6.86 ± 0.99; *P* < 0.001) than in the control group (3.62 ± 0.80). Also, the level of prostaglandin E_2_ was higher in LFA than in HFA (6.86 ± 0.99; *P* < 0.001).

**Table 3:** Level of corticosteroids, vanilyl mandelic acid, 5-hydroxyindoleacetic acid, and prostaglandin E_2_ in the urine of autistic children compared with age and sex matched children with typical development, shown as mean and standard deviation*^†^

Parameters	Control	LFA	MFA	HFA
**11- Hydroxycorticosteroids** **(μg cortisol/24 h)**	326.00 ± 63.57	649.15 ± 110.36^‡^	450.61 ± 56.33^‡^	377.76 ± 49.10^‡¶^
**Neutral 17- oxosteroids** **(mg/day)**	3.44 ± 0.74	10.68 ± 1.34^‡^	8.73 ± 1.48^‡^	4.07 ± 0.67^║¶^
**Total 17- oxosteroids** **(mg/day)**	21.45 ± 4.08	56.72 ± 9.36^‡^	48.01 ± 6.96^‡^	29.06 ± 4.07^‡¶^
**17- Oxogenic steroids** **(mg/day)**	18.01 ± 3.60	46.04 ± 5.99^‡^	39.28 ± 6.09^‡^	24.99 ± 3.87^‡¶^
**Total 17- Hydroxycorticosteroids** **(mg/day)**	15.39 ± 2.85	42.73 ± 6.62^‡^	33.82 ± 5.41^‡^	24.56 ± 3.81^‡¶^
**Vanilyl mandelic acid** **(mg/day)**	5.8 ± 1.28	38.99 ± 4.68^‡^	13.35 ± 1.74^‡^	7.56 ± 1.06^‡¶^
**5- Hydroxyindoleacetic acid** **(mg/day)**	8.2 ± 1.48	6.8 ± 0.85^‡^	7.4 ± 0.89^§^	7.8 ± 0.98^NS**^
**Prostaglandin E_2_** **(nmol/day)**	3.62 ± 0.80	11.41 ± 1.65^‡^	8.02 ± 1.12^‡^	6.86 ± 0.99^‡¶^

*Abbreviations: LFA – low functioning autism; MFA– moderately functioning autism; HFA– high functioning autism; NS – Non-significant.

†Variables were examined using one-way ANOVA and symbols represent significant differences from *post-hoc* Bonferroni test.

‡*P*< 0.001 (control vs LFA, MFA and HFA).

§*P* = 0.001 (control vs MFA).

║*P* = 0.006 (control vs HFA).

¶*P* < 0.001 (LFA vs HFA).

**P = 0.001 (LFA vs HFA).

Interestingly, Spearman’s rank correlation test showed that higher levels of corticosteroids (11-hydroxycorticosteroids vs CARS (r_s_: +0.795), neutral 17-oxosteroids vs CARS (r_s_: +0.852), total 17-oxosteroids vs CARS (r_s_: +0.902), 17-oxogenic steroids vs CARS (r_s_: +0.856), and total 17-hydroxycorticosteroids vs CARS (r_s_: +0.799), free cortisol vs CARS Phase I (r_s_: +0.899), Phase II (r_s_: +0.801), Phase III (r_s_: +0.787), vanilylmandelic acid vs CARS (r_s_: +0.834), and prostaglandin E_2_ vs CARS (r_s_: +0.876) were significantly positively correlated with the severity of autism (Table 2).

## Discussion

The present study found higher level of cortisol excretion in autistic children (LFA and MFA) than in the control group. Children with typical development showed a significantly lower cortisol excretion at noon time than LFA group.

The HPA axis, like most biological systems, is highly regulated and dependent on the ability of the system to maintain, respond to, and reset itself for homeostasis. Dysregulation of the HPA axis may manifest as disruptions in circadian rhythms, which in turn are represented by the pulsatile release of cortisol (24). A study in children with autism showed alterations in the normal circadian patterns of cortisol (25). The value of urinary free cortisol in assessing of adrenocortical function was first pointed out by Cope (26,27), who found that it detected increased adrenocortical function. Other studies also confirmed that an increase in the plasma concentration of free cortisol was accompanied by a linear increase in cortisol excretion in the urine (28). Urinary free cortisol in 24-hour samples has been widely used to assess basal cortisol secretion and has the theoretical advantage of being unaffected by possible cortisol circadian rhythm differences.

Normal physiological rhythms are responsible for all behavioral variables, including sleep organization and propensity, subjective alertness, and cognitive performance, which are disturbed in autistic children. There is increasing evidence to support the role of the sleep-wake cycle and the endogenous circadian system in the pathogenesis of major psychiatric disorders (29), and disturbed nocturnal sleep is a common observation among autistic children.

Our study also found a positive correlation between the elevated level of cortisol and the severity of autism. Cortisol secretion has earlier been shown to also markedly increase in response to stress, and autism has often been characterized as a disorder accompanied by increased arousal, stress, and sensory sensitivity (30-32). It was also shown that more severe autism led to more abnormal diurnal rhythm (33), which is in accordance with the present study.

Studies on major neurotransmitter systems (serotonin, catecholamine) strongly suggest that a major role in autism could be played by neurochemical factors (34). In the present study, urinary vanilyl mandelic acid, a marker of catecholamine metabolic status, was significantly higher in autistic children than in control group, which may be related to frequent stressful situations to which autistic children are subjected. The increased response to stressors could be due to worse handling of stress, over-elicitation of the physiological response, or dysfunctional stress response systems (35).

In this study, 5-hydroxyindoleacetic acid, a major metabolite of serotonin was significantly higher in LFA and MFA group and not significantly in HFA group than in the control group. Individuals with autism have been reported to have significantly higher serotonin (5-hydroxytryptamine) levels in whole blood and platelets (36). Though a higher level of serotonin has been reported in the blood of autistic children (37), the present study found significantly lower excretion level of 5-hydroxyindoleacetic acid in LFA and MFA group than in control group, which may be due to altered activity of monoamine oxidase, the enzyme responsible for oxidative deamination of serotonin to form the corresponding aldehyde, which is further oxidized to 5- hydroxyindoleacetic acid.

In the recent years, there has been a spate of research into the role of serotonin in neuropsychiatric conditions in childhood. Interestingly, Cohen et al (38) have reported lower concentrations of the serotonin metabolite 5-hydroxyindoleacetic acid in the cerebrospinal fluid in autistic than in non-autistic psychotic children. The interpretation of this finding is far from clear, however, neither group differed significantly from controls, whose concentration was between the autistic and psychotic group. Boullin et al (39) reported increased serotonin efflux as a finding specific to autism, but this was not confirmed by Yuwiler et al (40). Hence, the serotonin findings may well be important, but their meaning remains obscure. So far, attempts to relate serotonin concentration to clinical differences in autistic groups or groups with mental retardation have been rather disappointing (41).

Along with metabolic and rhythmic disturbances, there is emerging evidence of the contributing role of abnormal fatty acid metabolism in the pathology of autism (42). The present study showed a higher level of excretion of prostaglandin E_2_ in autistic children than in control group. There is evidence for increased prostaglandin metabolism in individuals with autism spectrum disorders (43). Eicosanoids, particularly prostaglandin D_2_ and E_2,_ are known to have sedative properties and to be involved in the control of the sleep-wake cycle (44), which was disturbed in autistic children in the present study.

There are reports (42) suggesting that fatty acid homeostasis may be altered in autism as a result of insufficient dietary supplementation, genetic defects, functional alteration of enzymes involved in their metabolism, or various environmental agents such as infections, inflammation, and drugs. Eicosanoids, derived from highly unsaturated fatty acids released from cell membranes by phospholipases and produced by cyclooxygenases, are required for normal functioning of synaptic junctions (45). Thus, any abnormality in phospholipase activity could result in alteration in neuronal structure and functions. Docosa-hexenoic acid and other free fatty acids can modulate abnormal electrical discharges from neurons (46) and a deficit of these fatty acids could increase susceptibility to epileptic seizures, which occurs in many patients with ASDs. In addition, the involvement of prostaglandin E_2_ signaling in early developmental process, including formation of dendritic spines and neuronal plasticity, is also emerging (47).

A major limitation of our study is that we did not determine the blood and saliva levels of corticosteroids, free cortisol, vanilyl mandelic acid, 5- hydroxyindole acetic acid, and prostaglandin E_2_, which still remains to be done, preferably in a study involving a greater number of children. Abnormal physiological rhythm found in autistic children should also be further investigated.

This study found that abnormal function of the HPA axis, evidenced by abnormal pattern of cortisol excretion in autistic children, could be strongly correlated with the severity of the disorder. Further studies on the factors responsible for abnormal circadian rhythm are needed, be it a gene, environmental stimuli, or an enzyme defect.
